# Increased ambient noise and elevated vocal effort contribute to airborne transmission of COVID-19

**DOI:** 10.1121/10.0002640

**Published:** 2020-11-30

**Authors:** Jonathan A. Kopechek

**Affiliations:** Department of Bioengineering, University of Louisville, Louisville, Kentucky 40292, USA

## Abstract

Widespread transmission of a novel coronavirus, COVID-19, has caused major public health and economic problems around the world. Significant mitigation efforts have been implemented to reduce the spread of COVID-19 but the role of ambient noise and elevated vocal effort on airborne transmission have not been widely reported. Elevated vocal effort has been shown to increase emission of potentially infectious respiratory droplets, which can remain airborne for up to several hours. Multiple confirmed clusters of COVID-19 transmission were associated with settings where elevated vocal effort is generally required for communication, often due to high ambient noise levels, including crowded bars and restaurants, meat packing facilities, and long-stay nursing homes. Clusters of COVID-19 transmission have been frequently reported in each of these settings. Therefore, analysis of COVID-19 transmission clusters in different settings should consider whether higher ambient noise levels, which are associated with increased vocal effort, may be a contributing factor in those settings. Mitigation strategies that include reduction of ambient noise, softer speech practices, and the use of technology such as microphones and speakers to decrease vocal effort will likely reduce the risk of transmitting COVID-19 or other airborne pathogens.

## BACKGROUND

The recent emergence of a new coronavirus, SARS-CoV-2, has caused widespread public health and economic challenges around the world. This coronavirus can spread very rapidly and cause COVID-19 disease, which is often associated with symptoms such as severe respiratory distress and pneumonia in many patients (Zhu *et al.*, 2020). According to the Johns Hopkins University Coronavirus Resource Center dashboard, as of October 2020 over 38 × 10^6^ people around the world have tested positive for COVID-19, including over 7 × 10^6^ in the United States, and over 1 × 10^6^ deaths have been attributed to COVID-19 globally (Dong *et al.*, 2020). To reduce transmission of COVID-19, many communities implemented significant mitigation measures, including school and business closures, travel restrictions, face mask mandates, and new social distancing requirements with a major emphasis on maintaining physical distancing of 6 ft or more. However, to date there has been very little guidance regarding the impact of ambient noise on COVID-19 transmission in public places. This analysis reviews the available evidence on this topic and suggests potential mitigation steps that may help businesses, schools, and other venues reduce transmission of COVID-19 or other airborne pathogens.

Airborne transmission of COVID-19 was suspected in the early stages of the outbreak based on experiences with other coronaviruses, including the 2003 outbreak of Severe Acute Respiratory Syndrome (SARS) and the emergence of Middle East Respiratory Syndrome (MERS) in 2012 (Yu *et al.*, 2004; Kim *et al.*, 2016). On January 10, 2020, the World Health Organization (WHO) recommended that health workers take precautions to protect against aerosolized respiratory droplets by implementing protective personal equipment (PPE) requirements in hospitals and medical centers. Recent studies have produced significant evidence that airborne transmission is a major factor, if not the primary factor, in COVID-19 outbreaks. For example, one study by Hamner *et al.* (2020) described high incidence of COVID-19 transmission associated with a choir practice in Skagit County, Washington, USA. A total of 61 people participated in a 2.5-h choir rehearsal on March 10, 2020, and physical contact was avoided by all attendees. However, 52 choir members became ill with COVID-19 symptoms (32 confirmed and 20 probable) within days of the choir practice. Point-source exposure is considered highly likely in this case based on the high attack rate (87%) and the timing of symptom onset in these patients. This cluster provides strong evidence that elevated vocal effort, such as occurs during singing, may increase risk of COVID-19 transmission.

Several recent studies have characterized respiratory droplets emitted during different types of speech, which are potential vectors of viral particles. Bourouiba (2020) reported that turbulent gas clouds, such as those produced by elevated exhalations, can entrap ambient air with clusters of respiratory droplets that evade evaporation much longer than isolated droplets (by up to a factor of 1000), extending the lifetime of these droplets from a fraction of a second to several minutes. In addition, this study reported that with optimal environmental conditions (e.g., temperature, humidity, etc.) a turbulent gas cloud could potentially spread virus-loaded respiratory droplets up to 23–27 ft away, well beyond the standard physical distancing guidelines of 6 ft. A separate study by Stadnytskyi *et al.* (2020) described additional characterization of the respiratory droplets emitted during speech by conducting empirical measurements of light scattering off the droplets. They found that loud speech could emit thousands of oral fluid droplets per second with a range of sizes, including droplets with diameters of 12–21 *μ*m that could remain airborne for up to 8–14 min. They calculated a 0.37% probability that a 10-*μ*m droplet contains at least one virion, which suggests that the risk of transmission is significantly increased during loud speech when large numbers of respiratory droplets are emitted. Asadi *et al.* (2019) reported that the rate of respiratory droplet emission is correlated with the loudness of vocalization, and some individuals are “speech superemitters” who consistently emit higher numbers of droplets compared to others. These studies show that loud speech and elevated vocal effort can cause increased emission of potentially infectious respiratory droplets, which is associated with increased risk of COVID-19 transmission.

These findings have important implications for settings with increased ambient noise where elevated levels of vocal effort are required for communication (Pearsons *et al.*, 1977). The WHO has reported that relaxed conversation is 100% intelligible with background noises below 35 dBA and fairly intelligible up to 45 dBA, but ambient noise levels above 65 dBA require increased vocal effort for conversation at a distance of 1 m (Berglund *et al.*, 1999). Prior studies have reported high ambient noise levels in many settings where COVID-19 outbreaks have been detected (Table I). A recent analysis by Courtemanche *et al.*, of social distancing impacts on COVID-19 transmission found that closing restaurant dining rooms and bars and/or entertainment centers/gyms led to statistically significant reductions in COVID-19 cases (Courtemanche *et al.*, 2020). Farber and Lily recently analyzed sound levels of restaurants and bars in New York City and found that a significant number of venues had high sound levels that were not conducive to conversation, which would require significant vocal effort (Farber and Wang, 2017). In their study over 71% of all restaurants and 90% of all bars exceeded 76 dBA, well above the threshold where increased vocal effort is required for conversation. Given that many customers at restaurants and bars are typically engaged in conversation at short distances, the high level of increased vocal effort could be an important factor in COVID-19 transmission at these locations. Lu *et al.* (2020) reported an outbreak of COVID-19 at a restaurant in Guangzhou, China, during late January–early February. A total of nine customers became infected with COVID-19, including several customers at separate tables spaced approximately 1 m apart. The authors report that 83 customers ate lunch at 15 tables in the same room as the infected persons. Although sound levels were not reported, with an average of 5.5 people per table and spacing of only 1 m between each table it is very likely that increased ambient noise levels in the restaurant caused significantly elevated vocal effort for conversation, which may have contributed to COVID-19 transmission in that setting.

**TABLE I.** Measured levels of ambient noise in various settings associated with clusters of COVID-19 transmission. The World Health Organization reports that ambient noise levels above 65 dBA require increased vocal effort for communication (Berglund *et al.*, 1999).

Setting	Noise level	Reference
Restaurants in New York City	71% of venues > 76 dBA	(Farber and Wang, 2017)
Bars in New York City	90% of venues > 76 dBA	(Farber and Wang, 2017)
Long-stay nursing homes	Average of 53–56 dBA (peak noise levels > 89 dBA)	(McCreedy *et al.*, 2018)
Meat packing facilities	Average of 71 dBA (peak noise levels > 100 dBA)	(Iulietto *et al.*, 2018)

Other settings have also been associated with increased transmission of COVID-19, and there appears to be a significant association between centers of outbreaks and areas of increased ambient noise and/or elevated vocal effort in most cases. Significant outbreaks of COVID-19 have been reported in senior living centers and nursing homes (McMichael *et al.*, 2020). It has been reported that more than 70% of long-stay nursing home residents have hearing loss, and more than half are moderately to severely impaired (McCreedy *et al.*, 2018). These levels of hearing loss likely contribute to elevated vocal effort in those environments. In addition, high ambient noise levels have also been reported in these settings from sources such as loud TVs or monitoring equipment, with mean noise levels of 53–59 dBA and peak noise volumes exceeding 89 dBA (Joosse, 2011). These levels often require increased vocal effort in the general population and would require even higher levels of vocal effort with residents who have hearing loss impairment.

Significant levels of COVID-19 transmission have also been reported in meat packing plants (Dyal *et al.*, 2020). In addition to reduced physical distancing in these settings, ambient noise levels are often significantly increased and elevated vocal effort is often required for communication. A recent smartphone app-based noise assessment in slaughterhouses found that average noise levels ranged from 71 dBA to over 100 dBA, well above the levels for soft speech to be intelligible (Iulietto *et al.*, 2018). Communication in these settings would require significant vocal effort at close distance, which increases the number of respiratory droplets emitted and likely contributes to the increased rates of COVID-19 transmission that have been detected in these types of facilities.

These studies reveal that high levels of vocal effort due to increased ambient noise could be an important factor in the airborne transmission of COVID-19 in settings where physical distancing is limited. Broad implementation of countermeasures to promote softer speech and reduce vocal effort could have a significant impact on decreasing emission of potentially infectious respiratory droplets. This has important implications for businesses, schools, and other venues that are implementing policies to reduce the risk of COVID-19 transmission in their facilities.

## CONCLUSIONS AND CONSIDERATIONS

There is substantial evidence that softer speech and decreased vocal effort may have an impact on reducing COVID-19 transmission. This suggests that settings with lower levels of ambient noise may have reduced risks of COVID-19 transmission. Strategies to reduce the risk of COVID-19 transmission may include community-based emphasis on approaches to decrease ambient noise exposures when feasible, combined with steps to promote softer speech practices and reduce vocal effort. Masks and face coverings can provide some protection by filtering infectious respiratory droplets in situations where vocal effort is required (Asadi *et al.*, 2020), and increasing air ventilation can reduce concentrations of potentially infectious airborne respiratory droplets (Milton, 2020), but other strategies to limit ambient noise and decrease vocal effort may also help mitigate the risk of COVID-19 transmission. Implementation of measures to reduce vocal effort in public settings and other places, such as nursing homes, may be beneficial. This may include increased adoption of technologies to assist verbal communication, such as microphones and speakers. Restaurants and bars may consider reducing or eliminating background music or other unnecessary sources of ambient noise in their premises when community spread of COVID-19 is elevated. Other work environments may also consider feasible options to muffle noise or implement other measures to reduce vocal effort required for communication. Additional studies are needed to better understand the impact of vocal effort on transmission of airborne pathogens in various settings, but the evidence described in this analysis indicates that efforts to decrease ambient noise and promote softer speech would likely reduce the transmission risk of COVID-19 or other airborne pathogens.
